# Genomic Characterization of New Variant of Hydrogen Sulfide (H_2_S)-Producing *Escherichia coli* with Multidrug Resistance Properties Carrying the *mcr-1* Gene in China †

**DOI:** 10.3390/antibiotics9020080

**Published:** 2020-02-13

**Authors:** Silpak Biswas, Mohammed Elbediwi, Guimin Gu, Min Yue

**Affiliations:** 1 Institute of Preventive Veterinary Sciences & Department of Veterinary Medicine, Zhejiang University College of Animal Sciences, Hangzhou 310007, China; silpakbiswas@gmail.com (S.B.); m.elbediwi@zju.edu.cn (M.E.); 2 Animal Health Research Institute, Agriculture Research Centre, Cairo 11435, Egypt; 3 Guangxi Center for Disease Prevention and Control, Nanning 530000, China; guguimin@163.com; 4 Zhejiang Provincial Key Laboratory of Preventive Veterinary Medicine, Hangzhou 310007, China

**Keywords:** antibiotic resistance genes, *Escherichia coli* variant, genome analysis, hydrogen sulfide, *mcr-1*

## Abstract

Colistin is considered to be a ‘last-resort’ antimicrobial for the treatment of multidrug-resistant Gram-negative bacterial infections. Identification of *Enterobacteriaceae*, carrying the transferable colistin resistance gene *mcr-1*, has recently provoked a global health concern. This report presents the first detection of a hydrogen sulfide (H_2_S)-producing *Escherichia coli* variant isolated from a human in China, with multidrug resistance (MDR) properties, including colistin resistance by the *mcr-1* gene, which could have great implications for the treatment of human infections.

## 1. Introduction

*Escherichia coli* is a significant cause of diseases in animals and humans worldwide [1], resulting in diverse community and hospital acquired infections, with major clinical concerns. Specific biochemical examinations, including the hydrogen sulfide (H_2_S) test, are important for identification of the *Enterobacteriaceae* species. The production of H_2_S, however, is not a typical characteristic of *E. coli*, though the H_2_S-producing variants of *E. coli* have also been reported previously [2,3]. Bacteria can produce H_2_S through orthologous enzymes, and recent studies have implicated H_2_S as a significant signaling molecule by protecting the bacteria from antibiotic-induced damage [4]. H_2_S can also prevent oxidative damage through stimulation of superoxide dismutase (SOD) and catalase activities [2,4]. Recent studies have demonstrated that H_2_S can also control the expression of *Staphylococcus aureus* virulence genes [5]. In this study, we present the characterization of a multidrug-resistant, H_2_S-producing *E. coli* isolated from the fecal sample from a clinically healthy patient in China.

## 2. Case Study

An active epidemiological surveillance study for foodborne pathogens was conducted towards healthy and diarrheal patients in Guangxi province, China. The initial aim was to screen *Salmonella* in the human fecal samples; we suspected this sample as *Salmonella*, and found this isolate was a lactose fermenter and H_2_S producer, according to a previous protocol [6]. To confirm whether this isolate was *Salmonella* or *E. coli,* we plated the sample on eosin methylene blue agar, and then confirmed the results with PCR identification and whole genome sequencing. Together, this is one isolate of interest, H_2_S-producing *E. coli* isolated from a 32-year old female from Guangxi province, China, during occupational health examination in 2015.

The isolate was sequenced using the MiSeq platform (Illumina Inc., San Diego, CA, USA), utilizing either 500 or 600 cycles of paired-end reads. The de novo assembly, using SPAdes 3.6, resulted in a genome size of 493,599 bp with GC content of 52.1%. The genome was annotated using the Rapid Annotation using Subsystem Technology (RAST) annotation server, and 1730 coding sequences (CDS) were identified. Detection of resistance genes and multilocus sequence typing (MLST) was accomplished at the Center for Genomic Epidemiology (CGE) (https://cge.cbs.dtu.dk/services/). We used the virulence factor database (VFDB) to obtain the virulence genes in this H_2_S-positive *E. coli* isolate. We performed antimicrobial susceptibility testing of the *E. coli* isolate using the broth microdilution method, as per the Clinical and Laboratory Standards Institute (CLSI) criteria [7]. The antimicrobials used are described in Table 1.

We found H_2_S-producing *E. coli* belonged to sequence type (ST) 10, serotype O10:H19, fimH25-fumC11 type. The typical virulence genes found in this *E. coli* isolate are shown in Table 2. The screening of the H_2_S-positive *E. coli* isolate for susceptibility to different antibiotics revealed that this H_2_S-positive variant was resistant to aminoglycosides, β-Lactams, polymyxins, fluoroquinolones, phenicols, sulfonamides, tetracyclines, and trimethoprim. Genome analysis revealed that this isolate also carried 3-mercaptopyruvate sulfurtransferase (*sseA*), indicating for the H_2_S production [4], and multiple antibiotic resistance (AR) genes. The conjugation assay confirmed both *sseA* and the *mcr* gene were on the chromosome. Table 1 shows the presence of AR genes for different antibiotics. Our study findings are clinically significant, highlighting the role of H_2_S as a microbial defense mechanism, revealing resistance against different clinically relevant antibiotics, including the ‘last-line’ therapeutic drug colistin, and also suggests the need of bacterial H_2_S inhibition in the treatment of infections caused by *E. coli*. The first extensive study of H_2_S-positive *E. coli* strains was found in Denmark [2]. Interestingly, it has been previously reported that H_2_S-generating enzymes (sseA in *E*. *coli*), especially, as mentioned, provided defense against antimicrobial compounds only in aerobic conditions [4]. The interesting point is that the cytoprotective effect of H_2_S is a universal defense mechanism found in bacteria as well as in mammals [2,4]. Moreover, the sequence type (ST) 10 *E. coli* strain is one of the predominant STs in the world [8].

We found aminoglycosides resistance genes *aadA*1, *aadA*2, trimethoprim resistance gene *dfrA12*, β-Lactams resistance gene *bla*_TEM-1B_, polymyxins resistance gene *mcr-1*, fluoroquinolones resistance genes *oqxA*, *oqxB*, phenicols resistance genes *floR, cmlA*1, sulfonamides resistance gene *sul3*, and tetracyclines resistance gene *tet*(A) in the H_2_S-positive *E. coli* isolate. The isolate was susceptible to carbapenems and cephalosporins (Table 1). The study by Jones et al. [3] and Harnett et al. [9] demonstrated previously that an H_2_S-producing variant of *Escherichia coli* isolated from a urinary tract infection (UTI) was also found to be resistant to different clinically relevant antibiotics. A previous study by Bailey et al. [1] reported the presence of *dfrA12*, *sul*3*, tet*(A), and *cmlA1,* including other AR genes in *E. coli* of healthy adults. It is interesting that *E. coli* of healthy humans represented a significant reservoir for several AR genes, as found in our study. The presence of *aadA*1, *aadA*2, and *dfrA12* genes were also reported previously in *E. coli* isolated from clinical samples in Malaysia [10]. Since the first report of colistin-resistant *E. coli* carrying the *mcr-1*-gene in China in 2016 [11], the existence and prevalence of the *mcr* gene and their variants has been reported in the *E.coli* across different continents. The bacterial cell membrane is the initial site of action for colistin. Colistin binds to lipopolysaccharide (LPSs) and phospholipids in the outer cell membrane of Gram-negative bacteria [12]. Colistin resistance facilitated by the mobile *mcr-1* gene has raised concerns during the last few years [13,14]. Fluoroquinolones (FQs), such as ciprofloxacin, have been the most commonly used antibiotics to treat UTIs caused by *E. coli*. However, the extensive use of fluoroquinolones has led to increasing fluoroquinolone resistance. The genes for multidrug efflux pump OqxAB, which are active on fluoroquinolones, were found for the first time in clinical isolates on a plasmid in *E. coli* in the USA in 2009 [15]. A recent study demonstrated the prevalence of plasmid-mediated quinolone resistance genes *oqxA* and *oqxB,* including other genes in clinical isolates of *E. coli,* obtained from UTIs in Azerbaijan and Iran [16]. Recently, *bla*_TEM-1B_ and *tet*(A) were found, including other AR genes in *E. coli* isolated from a patient in Lebanon, and linked to a bloodstream infection. Interestingly, previous studies reported that among *sul1*, *sul2,* and *sul3* genes responsible for sulfonamide resistance, both *sul1* and *sul2* are highly prevalent, and *sul3* has rarely been found [17,18]. Therefore, the presence of a rare sulfonamides resistance gene, *sul3,* could be an interesting characteristic of this H_2_S-producing *E. coli* strain. Antibiotic resistance genes found in this study were also reported previously in *E. coli* isolated from humans in various studies in Australia [19], Argentina [20], Tunisia [21], Croatia [22], Sweden [23], Spain [24], Bolivia and Peru [25], Algeria [26], Nigeria [27], and Lithuania [28].

## 3. Conclusions

This is the first report to describe H_2_S-producing colistin-resistant *E. coli* carrying the *mcr-1* gene, which also possesses the rare sulfonamide resistance gene *sul3*. The emergence and spread of the colistin resistance gene *mcr-1* in *E. coli* has attracted considerable attention worldwide. As endogenous H_2_S reduces the efficacy of many clinically used antimicrobials, the inhibition of this gas should be considered an effective therapy against a wide range of bacteria, including pathogenic *E. coli*. Continuous surveillance and molecular characterization of H_2_S-producing *mcr*-carrying *E. coli* is needed to shed light upon all of the transmission pathways. It is important to strengthen the hygiene practices in the hospital to reduce the environmental contamination by H_2_S-producing MDR *E. coli*. Our results require future extensive study and follow-up evaluations in order to understand the trends of the AR gene’s epidemiology in H_2_S-producing *E. coli*, in clinical settings and in the community, with time, and ultimately anticipate the detection of bacteria that can possibly cause serious public health concerns. In the future, it would be an interesting study to determine the H_2_S production by *E. coli*, in both aerobic and anaerobic conditions, to understand its contribution to antibiotic resistance. As a representative case, the H_2_S-producing *E. coli* isolate with AR genes observed in the study emphasizes the importance of rational use of antibiotics in future clinical practices.

## 4. Data Availability

Raw sequencing reads have been deposited in the NCBI BioProject database under accession number PRJNA576077.

## Figures and Tables

**Table 1:** Antibiotic phenotype with the corresponding resistance genes of H_2_S-producing *E. coli*.

Classes	Antibiotics	Minimum Inhibitory Concentration (MIC) Values (mg/L)	Interpretation	Antibiotic Resistance Genes
**Aminoglycosides**	Gentamicin	>32	R	*aadA*1, *aadA*2
	Kanamycin	64	R	
	Streptomycin	>64	R	
**β-Lactams**	Ampicillin	>128	R	*bla*_TEM-1B_
**Polymyxins**	Colistin	4	R	*mcr-1*
**Fluoroquinolones**	Ciprofloxacin	2	R	*oqxA*, *oqxB*
	Nalidixic acid	64	R	
**Phenicols**	Chloramphenicol	128	R	*floR, cmlA1*
**Trimethoprim /Sulfonamides/**	Trimethoprim/ Sulfamethoxazole	32/608	R	*dfrA12*, *sul3*
**Tetracyclines**	Tetracycline	>128	R	*Tet*(A)
**Carbapenems**	Imipenem	<0.5	S	
	Meropenem	0.5	S	
**Cephalosporins**	Cefotaxime	<0.5	S	
	Ceftiofur	<0.5	S	

R = Resistant; S = Susceptible.

**Table 2:** The virulence genes found in H_2_S-producing *E. coli* isolate.

Virulence Factors	Related Genes
**Adherence:**	
*E. coli* laminin-binding fimbriae (ELF)	*elfA*
*E. coli* laminin-binding fimbriae (ELF)	*elfC*
*E. coli* laminin-binding fimbriae (ELF)	*elfD*
*E. coli* laminin-binding fimbriae (ELF)	*elfG*
EaeH	*eaeH*
Hemorrhagic *E. coli* pilus (HCP)	*hcpA*
Hemorrhagic *E. coli* pilus (HCP)	*hcpB*
Type I fimbriae	*fimD*
Type I fimbriae	*fimF*
Type I fimbriae	*fimG*
Type I fimbriae	*fimH*
**Autotransporter:**	
Cah, AIDA-I type	*cah*
EhaB, AIDA-I type	*ehaB*
**Invasion:**	
Invasion of brain endothelial cells (Ibes)	*ibeB*
Invasion of brain endothelial cells (Ibes)	*ibeC*
**Non-LEE encoded TTSS effectors:**	
EspL1	espL1
EspL4	espL4
EspR1	espR1
EspR4	espR4
EspX4	espX4
**Secretion system** **:**	
ACE T6SS	aec15
ACE T6SS	aec17
ACE T6SS	aec18
ACE T6SS	aec19
ACE T6SS	aec22
ACE T6SS	aec24
ACE T6SS	aec25
ACE T6SS	aec26
ACE T6SS	aec27/ clpV
ACE T6SS	aec28
**Toxin** **:**	
Hemolysin/cytolysin A	hlyE/clyA
**Biofilm formation:**	
AdeFGH efflux pump/transport autoinducer	adeG
